# Comparison of the impact of *Tripterygium wilfordii* Hook F and Methotrexate treatment on radiological progression in active rheumatoid arthritis: 2-year follow up of a randomized, non-blinded, controlled study

**DOI:** 10.1186/s13075-018-1563-6

**Published:** 2018-04-10

**Authors:** Yang-zhong Zhou, Li-dan Zhao, Hua Chen, Yan Zhang, Dan-feng Wang, Lin-fang Huang, Qian-wen Lv, Bin Liu, Zhenbin Li, Wei Wei, Hongbin Li, Xiangping Liao, Hui Liu, Xiumei Liu, Hongtao Jin, Junxiang Wang, Yun-yun Fei, Qing-jun Wu, Wen Zhang, Qun Shi, Wen-jie Zheng, Feng-chun Zhang, Fu-lin Tang, Peter E. Lipsky, Xuan Zhang

**Affiliations:** 1 Department of Rheumatology and Clinical Immunology, Peking Union Medical College Hospital, Clinical Immunology Center, Chinese Academy of Medical Sciences and Peking Union Medical College, The Ministry of Education Key Laboratory, Beijing, 100730 China; 2 Department of Radiology, Peking Union Medical College Hospital, Chinese Academy of Medical Sciences and Peking Union Medical College, Beijing, China; 3 Formerly National Institute of Arthritis and Musculoskeletal and Skin Diseases, National Institutes of Health, Bethesda, MD USA; 4 Department of Rheumatology, The Affiliated Hospital of QingDao University Medical College, Qingdao, Shandong China; 5 Department of Rheumatology, The Bethune International Heping Hospital of Hebei, Shijiazhuang, Hebei China; 6 Department of Rheumatology, General Hospital of Tianjin Medical University, Tianjin, China; 7 Department of Rheumatology, Affiliated Hospital of Inner Mongolia Medical College, Hohhot, Inner Mongolia China; 8 Department of Nephrology and Rheumatology, The First People’s Hospital of ChenZhou, ChenZhou, Hunan China; 9 Department of Rheumatology, Beijing Dongfang Hospital, Beijing, China; 10 Department of Rheumatology, First Hospital of Shanxi Medical University, Taiyuan, Shanxi China; 11 Department of Rheumatology, Second Hospital of Hebei Medical University, Shijiazhuang, Hebei China; 12 Department of Rheumatology, Third Hospital of Hebei Medical University, Shijiazhuang, Hebei China; 13 The Body Sculpture and Liposuction Center of Plastic Surgery Hospital, Peking Union Medical College, Chinese Academy of Medical Sciences, Beijing, 100144 China; 14 AMPEL BioSolutions, Charlottesville, VA 22901 USA

**Keywords:** Rheumatoid arthritis, *Tripterygium wilfordii* Hook F, Radiological progression

## Abstract

**Background:**

*Tripterygium wilfordii* Hook F (TwHF) alone or in combination with methotrexate (MTX) has been shown to be more effective than MTX monotherapy in controlling the manifestations in subjects with disease-modifying antirheumatic drug (DMARD)-naïve active rheumatoid arthritis (RA) over a 6-month period. The long-term impact of these therapies on disease activity and radiographic progression in RA has not been examined.

**Methods:**

Patients with DMARD-naïve RA enrolled in the “Comparison of Tripterygium wilfordii Hook F with methotrexate in the Treatment of Active Rheumatoid Arthritis” (TRIFRA) study were randomly allocated into three arms with TwHF or MTX or the two in combination. Clinical indexes and radiographic data at baseline and year 2 was collected and compared using an intent-to-treat (ITT) and a per-protocol (PP) analysis. Two radiologists blinded to the treatment scored the images independently.

**Results:**

Of 207 subjects 109 completed the 2-year follow up. The number of subjects withdrawing from the study and the number adhering to the initial regimens were similar among the three groups (*p* > = 0.05). In the ITT analysis, proportions of patients reaching American College of Rheumatology 50% (ACR50) response criteria were 46.4%, 58.0% and 50.7% in the MTX, TwHF and MTX + TwHF groups (TwHF vs MTX monotherapy, *p* = 0.004). Similar patterns were found in ACR20, ACR70, Clinical Disease Activity Index good responses, European League Against Rheumatism good response, remission rate and low disease activity rate at year 2. The results of the PP analysis agreed with those in the ITT analysis. The changes in total Sharp scores and joint erosion and joint space narrowing during the 2 years were associated with changes in disease activity measured by the 28-joint count Disease Activity Score and were comparable among the three groups (*p* > 0.05). Adverse events were similar in the three treatment groups.

**Conclusions:**

During the 2-year therapy period, TwHF monotherapy was not inferior to MTX monotherapy in controlling disease activity and retarding radiological progression in patients with active RA.

**Trial registration:**

This is a follow-up study. Original trial registration: ClinicalTrials.gov, NCT01613079. Registered on 4 June 2012.

## Background

Rheumatoid arthritis (RA) is a chronic systemic autoimmune disease, characterized by synovitis, systemic inflammation and generation of autoantibodies. In industrialized countries, up to 1.0% of the adult population is affected by RA and suffers from joint damage and loss of physical function [1]. Disease-modifying antirheumatic drugs (DMARDs) are used to treat RA. The most commonly used DMARD is methotrexate (MTX), which is the “anchor drug” for active RA and can be combined with a variety of other drugs. Biological agents can be used when arthritis is aggressive and/or not sufficiently controlled by chemical DMARDs [2]. However, the world wide use of biological agents is restricted by their high costs and risk of severe infections [3].

*Tripterygium wilfordii* Hook F (TwHF) is widely used in traditional Chinese medicine as a potent treatment for joint pain, fever, chills, edema and local inflammation [4, 5]. Extracts of TwHF have been analyzed and the three major diterpenoids, triptolide, tripdiolide and triptonide, are mainly responsible for its anti-inflammatory and immune regulatory activities [6–9]. TwHF has been approved to treat RA in China. Our clinical experiences from treating more than 30,000 patients with RA each year in Peking Union Medical College Hospital (PUMCH) also support the high cost effectiveness of TwHF or a combination of MTX + TwHF, with increases in daily therapeutic expense less than 1 US dollar [10]. Previously in three randomized controlled trials, extracts of TwHF have also been shown to have good efficacy in treating RA compared with placebo or sulfasalazine [11–13].

To further evaluate the role of TwHF in treating RA by comparing its effects to MTX, we recently conducted the “Comparison of *Tripterygium wilfordii* Hook F with methotrexate in the treatment of active rheumatoid arthritis” (TRIFRA) study [14]. In this open-label, multicenter randomized controlled trial, 207 DMARD-naïve patients were randomly allocated into three arms and treated with TwHF, MTX or TwHF+MTX. We evaluated the proportion of patients achieving an American College of Rheumatology (ACR) 50% response (ACR50) at week 24, together with other parameters to measure disease activities including ACR20, ACR70, European League Against Rheumatism (EULAR) good or moderate response, clinical Disease Activity Index (cDAI), 28-joint count Disease Activity Score (DAS28), Health Assessment Questionnaire (HAQ) and 36-item Short-Form Health Survey questionnaire (SF-36) scores. At week 24, ACR50 response was achieved in about half of the patients using MTX or TwHF alone, and in more than three quarters of the patients receiving combination therapy. Similar patterns were found for other parameters. Moreover, with TwHF monotherapy and the combination therapy there was no increased incidence of adverse events compared to MTX alone. Thus, we concluded that TwHF monotherapy was not inferior to, and MTX + TwHF was better than, MTX monotherapy in controlling disease activity safely in patients with RA [14].

Long-term control of disease activity and associated joint damage, with the preservation of physical function in a safe manner is the ultimate goal of RA management. Therefore, the evaluation of treatment efficacy from long-term trials is needed to determine long-range benefit. Aside from benefit in clinical measures such as ACR50, prevention of joint damage evident on radiography (radiographic joint damage) is an important outcome in determining the long-term treatment effects in clinical trials, and recommended as a surrogate marker for overall functional status in patients with RA [15]. Previous studies have shown that treatment with MTX or other DMARDs could slow the progression of radiographic damage [16, 17]. The TRIFRA study was designed to be a 24-week, multicenter, randomized controlled trial (RCT). After its termination, the patients continued to be followed, and disease activities were monitored in the real-world situation. Based on changes in perceived disease activity, treatment could be modified accordingly. In this observational report, we followed the participants from the TRIFRA trial for 2 years after the study initiation. Both functional measures and radiological images were collected at year 2 to determine whether the same efficacy patterns observed in the first 24 weeks were sustainable. Moreover, the long-term impact on radiographic progression and physical function was determined.

## Methods

The TRIFRA study was designed as a 24-week, open-label, randomized study to evaluate the efficacy and safety of TwHF alone, MTX alone or the combination of TwHF + MTX in in the treatment of active RA. It was previously registered in ClinicalTrials.gov (NCT01613079). A detailed description of the study design was published previously [14]. After the end of the trial, subjects were followed by the investigators for 18 additional months and monitored using the same clinical outcome measures. In addition, radiographs of the hands and wrists were repeated to evaluate progressive joint damage.

### Patients

At the time of enrollment, patients eligible for this trial had to meet the following criteria: (1) 18–65 years of age; (2) diagnosed with RA as determined by meeting the 2010 ACR/EULAR classification criteria and having had RA for at least 6 weeks; (3) at least three swollen joints (swollen joint count (SJC)) and five tender joints (tender joint count (TJC)); (4) erythrocyte sedimentation rate (ESR) >28 mm/h or C-reactive protein (CRP) >20 mg/L. Patients who completed 24 weeks in the TRIFRA study continued into the next 18 months of follow up. All patients signed written informed consent at the time of enrollment.

### Study protocol

**Fig. 1:**
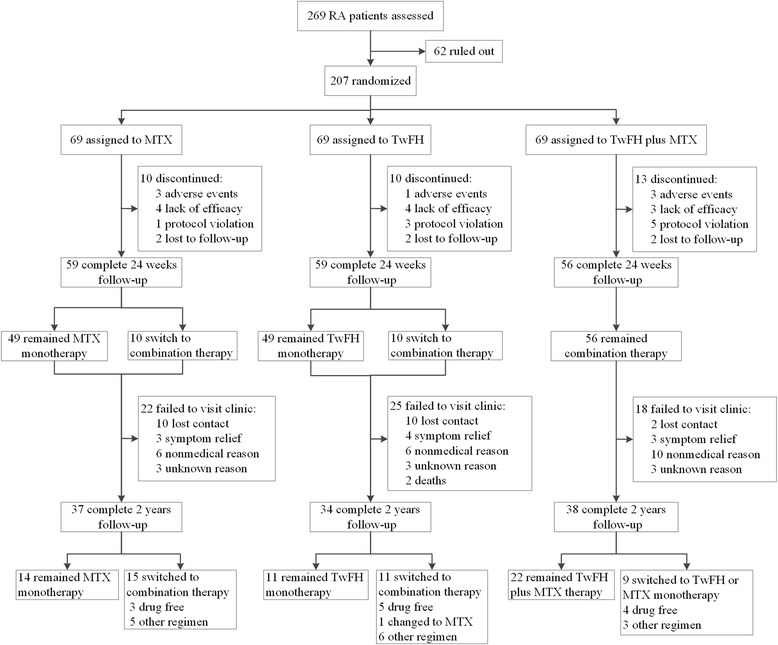
Study design and numbers of patients in each group who completed or withdrew from the 24-week TRIFRA study and 2-year follow up. RA, rheumatoid arthritis; MTX, methotrexate; TwHF, *Tripterygium wilfordii* Hook F

The protocol was approved by Peking Union Medical College Hospital (PUMCH) ethical review board. Initially, enrolled participants were allocated into three arms by centralized randomization, as follows: oral TwHF pills 20 mg three times a day; MTX starting from 7.5 mg once a week and increasing to 12.5 mg once a week (0.20–0.25 mg/kg) within 4 weeks, with folic acid 10 mg on the day after each MTX administration; or TwHF plus MTX at the same dosage as aforementioned. In this study, the TwHF used was the same as that in the TRIFRA study, in which the concentration of triptolide (C20H24O5), the major immunosuppressive anti-inflammatory diterpenoid, was 1.2 μg/10 mg, and the concentration of wilforlide (C30H46O3), an anti-inflammatory triterpene, was 36.6 μg/10 mg. The patient’s DAS28 was evaluated at week 12, and monotherapy was continued only if their DAS28 reduced more than 30%; otherwise the patients switched to MTX + TwHF combination therapy. After 24 weeks, patients were followed up and their therapy would be modified based on the physician’s judgement. A detailed description of the study design was published previously and is also shown in Fig. 1.

### Outcomes and measurements

Similar to the initial 24-week TRIFRA study, though the treating doctors and patients were not blinded to medication allocation, a similar set of clinical efficacy parameters were evaluated in each patient at the end of the second year by trained evaluators who were unaware of the specific therapeutic regimen. The parameters included the ACR criteria [18], HAQ [19], the ESR or serum CRP level, EULAR good or moderate response, cDAI good response (defined as achieving ≥ 50% improvement in the cDAI, or cDAI ≤2.8) [20], clinical remission (defined as DAS28 <2.6) and low disease activity (LDA) (defined as DAS28 <3.2) [21] and change in HAQ or 36-item Short-Form Health Survey questionnaire (SF-36) scores. The proportion of patients achieving 20% improvement using the ACR criteria was calculated as ACR20, and similarly, the ACR50, and ACR70 were calculated. The safety profile was also recorded.

During the 2 years, radiographic progression was analyzed in patients who had at least two radiographic examinations with time intervals longer than 1 year. Radiographic images of the hands and wrists were independently read by two radiologists who were masked to treatment allocation, time sequence of radiographs and the patient’s clinical response. Joint erosions (JE) and joint space narrowing (JSN) were scored, which were summed to calculate the modified total Sharp score (mTSS) [22]. Inter-reader variability was assessed by the intraclass correlation coefficient and based on status score it ranged from 0.794 to 0.907. To balance the time-interval difference, linear extrapolation of actual change from baseline images was used for patients whose image was missing at the 2-year time point. Mean scores of the two radiographic readers were used for analysis. The radiographic data were reported in a systematic way as recommended [23]. mTSS non-progression was defined as a change from baseline mTSS between − 0.5 ~ 0.5 units at 2 years or less than the smallest detectable difference (SDD) [24]. The SDD was computed based on the observed difference between the readers. The estimated yearly mTSS progression at the baseline was defined as the baseline mTSS score divided by disease duration for each patient.

### Statistical analysis

Analysis was performed using the modified intent-to-treat (ITT) method, which included all the patients who received the originally allocated treatment at least once. This method was used for the analysis of ACR responses, cDAI responses, EULAR responses, DAS28 remission, ESR, high sensitivity (hs)CRP level, pain measured on a visual analog scale (VAS) and HAQ score at year 2, with missing data interpolated with the last observation carried forward (LOCF) approach. To compare the efficacy variables of MTX monotherapy and TwHF monotherapy, a non-inferiority test was carried out. In the TRIFRA study, the non-inferiority margin was set as 10%, and the required sample size was then calculated accordingly with at least 80% power and 5% level of significance [14]. In this follow-up study, we used the same non-inferiority margin as we did in the TRIFRA study, as the sample size in the ITT analysis was the same. The efficacy variables were compared in the MTX + TwHF group and the MTX monotherapy using the chi square (χ^2^) test. We also conducted a per-protocol (PP) analysis that only included the participants who finished the 2-year follow up without violating the originally allocated treatment regimen.

A valid-for-efficacy (VFE) analysis was conducted for radiographic data, including patients who completed the 2-year follow up (completers). Radiographic changes in mTSS, JE and JSN scores were analyzed using analysis of covariance (ANCOVA) with treatment and baseline scores as covariates. Only patients with baseline images and at least one radiographic assessment after the initiation of treatment were included in the analysis. Radiographic non-progression was defined as an absolute value of the change in mTSS no greater than 0.5, which was analyzed using the χ^2^ test.

Categorical data are presented as number (n) or percentage (%). Continuous data are presented as mean (SD) or median (25th–75th centiles). Differences between groups were analyzed for significance using the χ^2^ test (categorical data) or ANCOVA with factors for treatment and baseline scores as covariates (continuous data). All analyses were computed using SPSS statistics V.22.0and SAS V.9.1.

## Results

### Patients’ follow up and withdrawal information

A total of 207 patients participated in the TRIFRA study. All three treatment groups were well-balanced with respect to baseline demographic and clinical characteristics [14]. Among 207 recruited patients, 33 patients (16%) dropped out in the first 24 weeks mainly because of side effects, inefficacy and protocol violation (Fig. 1). After that, 22 patients (11%) were lost to follow up and 41 patients (20%) refused to return for disease evaluation at year 2. Reasons for refusal included unwillingness because of symptom relief (10/41, 24%), non-medical reasons (22/41, 54%) and unwillingness to disclose information (9/41, 22%). The non-medical reasons mainly include travel expenditure and other personal issues. Two patients died of malignancy. Among 207 recruited patients, a total of 109 patients (53%) returned for the 2-year follow-up evaluation.

As shown in Fig. 1, numbers of patients who withdrew from the study at year 2 were comparable among the MTX monotherapy group (32/69, 46.4%), the TwHF monotherapy group (35/69, 50.7%) and the combination therapy group (31/69, 44.9%) (*p* = 0.777), and the rate of maintaining the initial protocol was not significantly different among the three groups (14/69 (20.3%) in the MTX group, 11/69 (15.9%) in the TwFH group and 22/69 (31.9%) in the combination group, *p* = 0.069). However, there was a trend towards a higher compliance rate among patients in the combination group compared to the other two groups. As aforementioned, the numbers of patients with favorable outcomes (including patients maintaining the initial protocol, patients no longer taking any drugs and patients declining to return for evaluation because of symptom relief) were not significantly different (20/69 (29.0%) in the MTX group, 20/69 (29.0%) in the TwFH group and 29/69 (42.0%) in the combination group, *p* = 0.172), although there was also a trend towards a higher favorable outcome rate among patients in the combination group compared to the other two groups.

### Clinical efficacy

#### Disease activity evaluation

Disease activity was evaluated at year 2 by the ACR criteria, cDAI, EULAR good response, remission rate (DAS28 <2.6) and LDA rate (DAS28 <3.2). In the ITT analysis, we performed a non-inferiority test to compare the TwHF monotherapy group and the MTX monotherapy group, and similar statistical significances were shown in all the parameters: ACR20, 73.9% vs 55.0%; ACR50: 58.0% vs 46.4%; ACR70: 34.8% vs 21.7%; cDAI good response: 72.5% vs 56.5%; EULAR good response, 47.8% vs 23.2%; remission rate, 43.5% vs 17.4%; and LDA rate, 47.8% vs 26.1% (*p* < 0.05) in the TwHF vs MTX group (Table 1). There was a similar pattern at week 24 as described previously [14]. When we compared disease activity in patients from the combination and MTX monotherapy groups, there were significant differences in ACR20, EULAR good response and DAS remission rate at year 2 (ACR 20, 72.5% vs 55.0%; EULAR good response, 40.6% vs 23.2%; remission rate, 34.8% vs 17.4%, respectively (*p* < 0.05) (Table 1).

**Table 1:** Clinical efficacy measures over 2 years in intention-to-treat (ITT) analysis and per-protocol (PP) analysis

Variables	Year 2									
	ITT					PP				
	MTX (*n* = 69)	TwFH (*n* = 69)	TwFH vs MTX	TwFH + MTX (n = 69)	TwFH + MTX vs MTX	MTX (*n* = 14)	TwFH (*n* = 11)	TwFH vs MTX	TwFH + MTX (*n* = 22)	TwFH + MTX vs MTX
ACR20 response, *n* (%)	38 (55.0%)	51 (73.9%)	*p* < 0.001	50 (72.5%)	*p* = 0.034	8 (57.1%)	10 (90.9%)	*p* < 0.001	14 (63.6%)	*p* = 0.163
ACR50 response, *n* (%)	32 (46.4%)	40 (58.0%)	*p* = 0.005	35 (50.7%)	*p* = 0.609	7 (50.0%)	8 (72.7%)	*p* < 0.001	11 (50.0%)	*p* = 0.312
ACR70 response, *n* (%)	15 (21.7%)	24 (34.8%)	*p* = 0.001	20 (29.0%)	*p* = 0.328	4 (28.6%)	4 (36.4%)	*p* = 0.013	7 (31.8%)	*p* = 0.346
cDAI response, *n* (%)	39 (56.5%)	50 (72.5%)	*p* = 0.001	37 (53.6%)	*p* = 0.732	8 (57.1%)	9 (81.8%)	*p* < 0.001	15 (68.2%)	*p* = 0.110
EULAR good response, *n* (%)	16 (23.2%)	33 (47.8%)	*p* < 0.001	28 (40.6%)	*p* = 0.028	5 (35.7%)	7 (63.6%)	*p* < 0.001	9 (40.9%)	*p* = 0.259
EULAR good to moderate response, *n* (%)	50 (72.5%)	57 (82.6%)	*p* = 0.002	59 (85.5%)	*p* = 0.060	12 (85.7%)	10 (90.9%)	*p* = 0.003	21 (95.5%)	*p* = 0.072
DAS28 remission, *n* (%)	12 (17.4%)	30 (43.5%)	*p* < 0.001	24 (34.8%)	*p* = 0.020	4 (28.6%)	7 (63.6%)	*p* < 0.001	7 (31.8%)	*p* = 0.346
DAS28 remission and LDA, *n* (%)	18 (26.1%)	33 (47.8%)	*p* < 0.001	28 (40.6%)	*p* = 0.071	5 (35.7%)	7 (63.6%)	*p* < 0.001	8 (36.4%)	*p* = 0.784

*MTX* methotrexate, *TwHF Tripterygium wilfordii* Hook F, *ACR* American College of Rheumatology, *cDAI* clinical Disease Activity Index, *EULAR* European League Against Rheumatism, *DAS28* 28-joint count Disease Activity Score, *LDA* low disease activity

The number within each bracket represents the patients who reached the response criteria in each group. The percentage of response was calculated with the denominator of total enrolled patients (69 for each group) in the ITT analysis or patients who finished the originally allocated treatment in the PP analysis (14, 11 and 22 for the MTX, the TwHF and the combination groups, respectively). In the ITT analysis, those patients who withdrew from the trial prematurely or switched to the combination group were classed as missing data, which were calculated using the last observation carried forward imputation method when performing the ITT analysis

The *p* values for comparison between the MTX group and the TwHF group were calculated using the non-inferiority test. Comparison of the combination treatment and MTX monotherapy was calculated using the χ2 test

We also carried out the PP analysis that only included the patients who followed the allocated treatment regimen for 2 years. The results agreed with those found in the ITT analysis. The non-inferiority test was used to compare the TwFH group and MTX group, and showed statistical significances in all the parameters at year 2. However, there was no significant difference between the combination therapy and MTX monotherapy groups (Table 1).

We compared the core components of ACR responses and DAS28 in the three groups (Table 2). All treatment groups had decreases in DAS28 and HAQ scores and increases in SF36 scores, suggesting improvement in functional disability and life quality. However, there was no statistically significant difference in the improvement of these scores among the three groups at year 2 (*p* > 0.05) in either the ITT or the PP analysis (Table 2).

**Table 2:** Clinical and laboratory measures in the three groups over 2 years in intention-to-treat (ITT) analysis and per-protocol (PP) analysis

Variables	Baseline			Year 2							
				ITT				PP			
	MTX (n = 69)	TwFH (n = 69)	TwFH + MTX (n = 69)	MTX (n = 69)	TwFH (n = 69)	TwFH + MTX (n = 69)	*P*	MTX (n = 14)	TwFH (n = 11)	TwFH + MTX (n = 22)	*P*
ESR (mm/h)	54.0 (28.0)	45.1 (24.6)	51.6 (25.9)	27.7 (22.6)	21.9 (17.9)	26.7 (27.3)		20.6 (16.8)	19.8 (19.0)	22.7 (26.4)	
mean (SD)	54.0 (28.3, 77.0)	38.0 (26.0, 67.0)	48.2 (31.0, 72.0)	23.0 (10.0, 34.8)	15.0 (9.0, 31.0)	17.5 (9.0, 34.8)	0.760	17.0 (8.0, 27.3)	12.0 (5.0, 28.0)	15.5 (6.0, 28.5)	0.868
median (IQR)											
CRP (mg/L)											
mean (SD)	37.5 (40.4)	27.3 (25.0)	30.1 (28.7)	20.6 (34.6)	11.3 (20.7)	14.7 (28.6)		9.0 (18.4)	15.2 (37.7)	9.8 (20.3)	
median (IQR)	21.7 (8.0, 53.8)	17.9 (7.0, 38.4)	24.8 (9.5, 42.6)	5.9 (1.4, 20.0)	3.5 (1.0, 12.7)	4.0 (2.0, 19.4)	0.803	1.7 (0.7, 9.4)	2.4 (0.7, 5.6)	3.7 (1.2, 9.1)	0.375
VAS (mm)											
mean (SD)	71.4 (22.4)	68.5 (25.5)	70.5 (21.3)	36.8 (28.7)	32.1 (27.5)	36.7 (27.4)		33.2 (31.0)	29.1 (30.8)	33.6 (25.5)	
median (IQR)	72.5 (50.0, 90.0)	70.0 (50.0, 95.0)	75.0 (50.0, 85.0)	30.0 (10.0, 57.5)	20.0 (10.0, 50.0)	32.5 (15.0, 60.0)	0.674	30.0 (7.5, 55.0)	20.0 (0, 50.0)	32.5 (7.5, 60.0)	0.966
PGA (mm)											
mean (SD)	68.0 (24.6)	65.5 (23.6)	65.8 (21.3)	37.7 (27.5)	32.7 (27.6)	38.8 (25.9)		33.6 (27.6)	21.8 (24.4)	35.7 (25.7)	
median (IQR)	70.0 (50.0, 87.5)	60.0 (50.0, 87.5)	62.5 (50.0, 80.0)	30.0 (10.0, 57.5)	25.0 (10.0, 50.0)	40.0 (18.8, 60.0)	0.959	30.0 (10.0, 50.0)	20.0 (0, 30.0)	37.5 (10.0, 60.0)	0.373
PhGA (mm)											
mean (SD)	62.8 (23.5)	60.0 (23.7)	60.7 (23.1)	34.2 (27.7)	31.0 (26.6)	34.8 (26.1)		31.4 (32.3)	26.4 (26.9)	34.5 (26.8)	
median (IQR)	65.0 (43.5, 80.0)	60.0 (40.0, 80.0)	60.0 (45.0, 80.0)	30.0 (10.0, 60.0)	20.0 (10.0, 50.0)	30.0 (10.0, 51.3)	0.826	30.0 (3.8, 55.0)	20.0 (0, 50.0)	32.5 (10.0, 60.0)	0.701
TJC											
mean (SD)	16.2 (8.3)	13.8 (7.2)	15.5 (8.3)	6.9 (7.1)	4.5 (5.8)	6.3 (7.5)		6.1 (6.3)	2.5 (3.4)	0.9 (1.4)	
median (IQR)	16.0 (7.5, 23.5)	13.0 (7.5, 17.0)	14.0 (8.0, 22.3)	5.0 (2.0, 10.0)	2.0 (0, 7.0)	3.0 (1.0, 9.0)	0.287	3.0 (1.0, 10.5)	2.0 (0, 5.0)	0 (0, 2.3)	0.368
SJC											
mean (SD)	9.1(6.0)	8.9 (6.1)	8.9 (6.7)	2.9 (4.4)	2.5 (4.3)	2.4 (4.6)		3.1 (4.5)	0.3 (0.6)	2.2 (4.2)	
median (IQR)	7.0 (4.5, 13.0)	7.0 (4.0, 12.0)	7.0 (3.0, 11.3)	2.0 (0, 4.0)	0 (0, 4.0)	0 (0, 3.0)	0.836	1.0 (0, 5.0)	0 (0, 0)	0 (0, 2.3)	0.191
HAQ											
mean (SD)	1.5 (0.9)	1.3 (0.9)	1.4 (0.9)	1.2 (3.6)	0.5 (0.7)	0.6 (0.7)		2.6 (7.9)	0.4 (0.6)	0.6 (0.6)	
median (IQR)	1.5 (0.9, 2.3)	1.3 (0.6, 2.0)	1.5 (0.6, 2.1)	0.5 (0, 1.3)	0.1 (0, 0.9)	0.4 (0, 1.0)	0.162	0.1 (0, 0.9)	0(0, 0.9)	0.5 (0.1, 1.1)	0.286
SF36											
mean (SD)	237.8 (127.0)	292.6 (155.3)	279.1 (153.4)	388.8 (138.4)	448.9 (152.8)	417.2 (146.9)		453.3 (78.3)	469.7 (70.4)	445.1 (74.6)	
median (IQR)	231.0 (143.8, 310.0)	269.3 (181.2, 406.4)	260.5 (163.5, 364.3)	397.5 (305.5, 489.5)	470.2 (351.3, 536.5)	411.8 (329.8, 507.3)	0.259	478.1 (373.6, 525.7)	493.3 (426.8, 509.7)	460.8 (397.8, 506.8)	0.609
DAS28											
mean (SD)	6.62 (1.31)	6.22 (1.28)	6.38 (1.40)	4.16 (1.70)	3.54 (1.69)	3.95 (1.77)		3.82 (1.96)	2.83 (1.72)	3.58 (1.63)	
median (IQR)	6.66 (5.48, 7.60)	6.16 (5.37, 7.06)	6.26 (5.47, 7.37)	3.70 (3.05, 5.12)	3.49 (2.09, 4.86)	3.89 (2.51, 5.13)	0.298	3.45 (2.71, 5.52)	2.40 (1.42, 4.49)	3.53 (2.01, 4.63)	0.613

*ESR* erythrocyte sedimentation rate, *CRP* C-reactive protein, *PGA* Patient’s global assessment of disease activity, *PhGA* Physician’s global assessment of disease activity, *TJC* tender joint count, *SJC* swollen joint count, *HAQ* Health Assessment Questionnaire, *SF-36* 36-item Short-Form Health Survey questionnaire, *DAS28* 28-joint count Disease Activity Score, *MTX* methotrexate, *TwHF Tripterygium wilfordii* Hook F

Data are presented as mean (standard deviation, SD) and median (interquartile range, IQR)

The *p* values were calculated using the χ2 test

### Radiographic outcome

At year 2, paired evaluable radiographic results before and after treatment initiation were available in 109 patients for the VFE analysis (52.7%). Mean TSS at baseline were 28.15, 33.02 and 26.8 in the MTX, TwHF and combination therapy groups, respectively (*p* = 0.768) (Table 3). After 2 years, the mean change from baseline in JE, JSN and TSS were not significantly different among the three treatment groups (*p* > 0.05). The estimated annual radiographic progression was lower in the TwHF and combination group compared with the MTX group. However, there was no statistical significance on ANCOVA with the baseline score as a covariate (*p* = 0.615).

**Table 3:** Radiographic changes from baseline after 2 years of treatment

	MTX (*n* = 37)	TwHF (*n* = 34)	MTX + TwHF (*n* = 38)	*P*
JE at baseline (range 0–280)				
mean (SD)	16.76 (22.56)	20.83 (29.02)	14.92 (21.26)	
median (IQR)	7.25 (2.00, 25.00)	7.00 (2.75, 27.00)	5.00 (2.75,15.50)	0.589
JSN at baseline (range 0–168)				
mean (SD)	11.92 (15.35)	13.05 (16.18)	12.34 (16.06)	
median (IQR)	6.25 (0, 17.50)	3.00 (0.75, 30.75)	5.00 (1.25, 21.00)	0.957
TSS at baseline				
mean (SD)	28.68 (35.54)	33.88 (42.97)	26.99 (35.81)	
median (IQR)	14.75 (2.75, 42.13)	15.00 (5.00, 47.00)	14.5 (5.00, 35.25)	0.738
Estimated annual radiographic progression at baseline^†^				
mean (SD)	20.96 (78.12)	13.02 (15.07)	10.84 (15.90)	
median (IQR)	5.74 (1.88, 10.21)	6.25 (3.51, 20.17)	5.61 (2.25, 10.37)	0.635
mTSS at 2 years				
mean (SD)	31.94 (37.54)	36.57 (44.04)	30.21 (35.99)	
median (IQR)	15.25 (3.94, 42.00)	18.00 (5.50, 51.67)	15.71 (7.41, 38.81)	0.826
ΔmJE				
mean (SD)	1.59 (4.43)	1.33 (2.03)	1.44 (3.22)	
median (IQR)	0 (0, 1.21)	0.75 (0, 2.32)	0.77 (0, 1.50)	0.939
ΔmJSN				
mean (SD)	1.67 (3.32)	1.31 (2.41)	1.78 (3.24)	
median (IQR)	0 ( 0, 1.13)	0 (0, 1. 52)	0 (0, 2.19)	0.781
ΔmTSS				
mean (SD)	3.24 (6.95)	2.70 (3.70)	3.22 (5.66)	
median (IQR)	0.61 (0,4.18)	1.00 (0,4.14)	1.04 (0,3.62)	0.862
ΔmTSS <0.5	17 (45.95%)	12 (35.29%)	13 (34.21%)	0.520
ΔmTSS ≤SDD (5.24)	31 (83.78%)	27 (79.41%)	31 (81.58%)	0.893
ΔmJE ≤SDD (3.37)	32 (86.49%)	31 (91.18%)	35 (92.11%)	0.691
ΔmJSN ≤SDD (2.85)	30 (81.08%)	27 (79.41%)	31 (81.58%)	0.955

*MTX* methotrexate, *TwHF Tripterygium wilfordii* Hook F, *JE* joint erosion, *JSN*joint space narrowing, *TSS* total Sharp score, *mTSS* modified total Sharp score, *SSD* smallest detectable difference

Data are presented as mean (standard deviation, SD) and median (interquartile range, IQR)

The *p* values were calculated using analysis of variance for baseline scores, while comparison for estimated progression and scores at year 2 was performed using analysis of covariance with baseline score as a covariate. Comparison of the change in score <0.5 or equal to or less than the smallest detectable difference was conducted using the χ2 test

^†^Estimated annual radiological progression at baseline was defined as the baseline mTSS score divided by disease duration for each patient

After 2 years, 34.21% of patients receiving combination therapy had no radiographic progression (change from baseline in the mTSS <0.5), compared with 35.29% of patients receiving TwHF and 45.95% of those receiving MTX (*p* = 0.520). In the majority of patients (81.58% of those receiving combination therapy, 79.41% of those receiving TwHF and 83.78% of those receiving MTX), the change in the TSS was equal to or less than the SDD (5.24 units) (*p* = 0.893). Similarly, when JE and JSN were evaluated independently, changes equal to or less than the SDD were observed in the majority of patients in the three groups (*p* > 0.05).

**Fig. 2:**
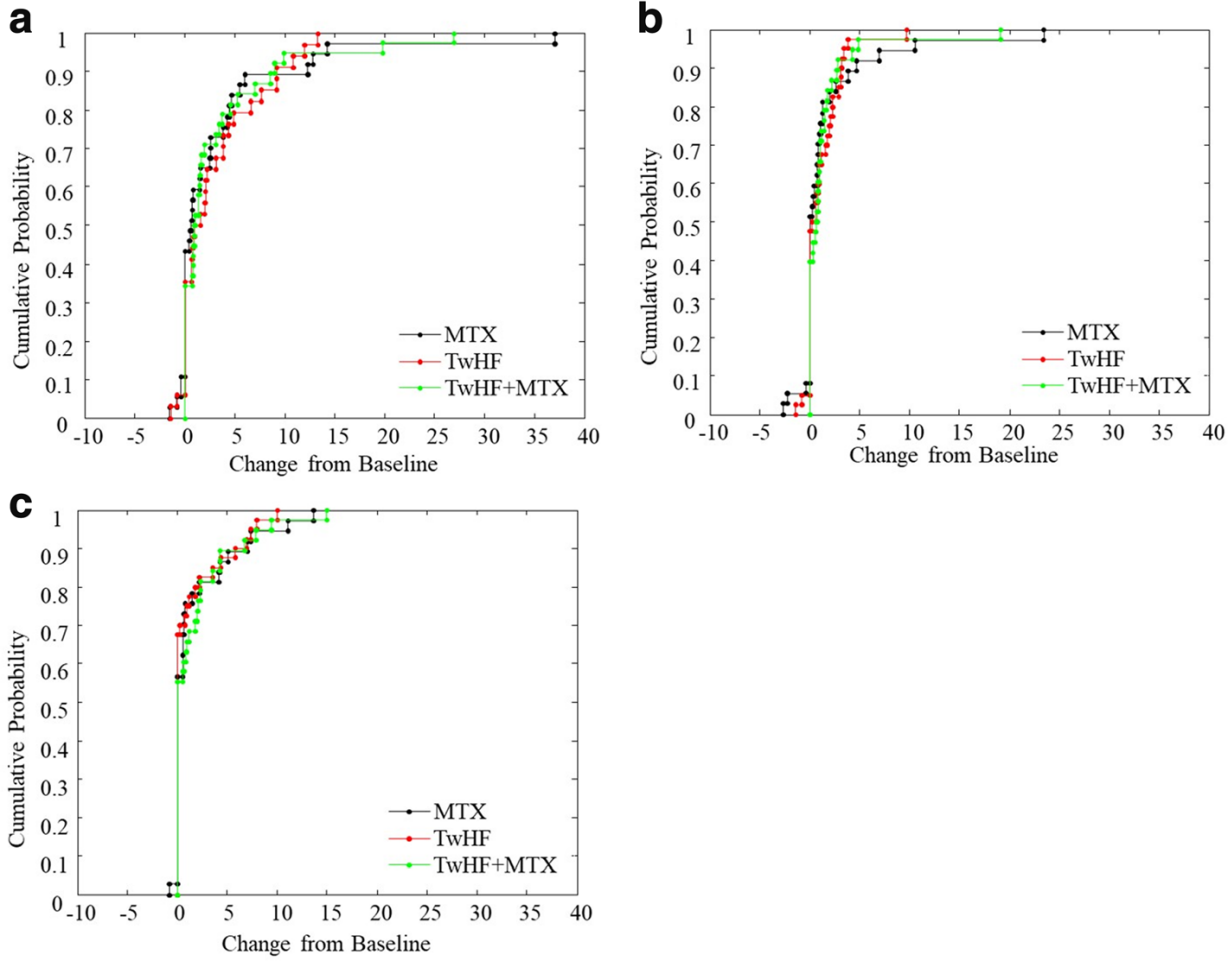
Cumulative probability distribution for the modified total Sharp scores (**a**), joint erosions (**b**), and joint space narrowing (**c**) over the 2 years. MTX, methotrexate; TwHF, *Tripterygium wilfordii* Hook F

The changes from baseline in TSS, JE and JSN scores were presented in cumulative probability plots to visualize radiographic data in all three groups (Fig. 2), and the majority of observations in the three treatment groups had values close to zero. The plots presenting change within the three groups were similar, indicating a comparable change in radiographic damage. The association between JE/JSN progression and disease activity has been reported before [25]. In our data, we analyzed the association between radiological progression and tertiles of change in the DAS28 during the 2 years. The increasing tertiles of change in the DAS28 were associated with JE, JSN and mTSS progression when analyzed with treatment as a covariate (*p* < 0.05).

### Side effects

Adverse events were monitored during the 2 years. Overall, 54.6% of the patients reported adverse events, 65% in the MTX, 48% in the TwFH and 51% in the combination group (*p* = 0.089) (Table 5). Similar to previous reports, the most common adverse effects recorded were gastrointestinal, including nausea, abdominal discomfort and liver dysfunction. Serious infection, such as pneumonia and urinary tract infection, were reported in five patients in the MTX group and two patients in the combination group. Two deaths from malignancy were reported; one subject died of gastric cancer and the diagnosis was unclear in the other subject. Among 170 female patients, 101 were postmenopausal and 17 (10.0%) developed irregular menstruation during the 2-year follow up, including 5 in the MTX group, 7 in the TwHF group and 5 in the combination group (*p* = 0.744).

In this follow-up study, none of the patients was reported to discontinue the treatment because of adverse events in any of the three arms. Separately, we also compared the side effects reported by the 109 patients who completed the 2-year follow up. Overall, 36.7% of the patients reported adverse events. Among these patients, the most common adverse effects were nausea and liver function abnormalities. Serious infection was not reported in any of the groups.

## Discussion

The previous 24-week TRIFRA clinical trial is the first RCT that compared the efficacy of TwHF and MTX in treating DMARD-naïve patients with RA [14]. At week 24, TwHF was not inferior to MTX as measured by multiple parameters of disease activity, including ACR20, ACR50 and ACR70 response criteria, EULAR and cDAI good response criteria and DAS28 remission criteria and LDA rate. More importantly, patients with RA receiving MTX + TwHF combination therapy had better improvement in disease activity. Considering that RA is a chronic disease, we followed up the patients from the TRIFRA trial at year 2 and evaluated disease activity in the same way. Among 207 patients recruited in the TRIFRA trial, 109 of them returned for the 2-year follow up. Notably, the frequency of adhering to the initial protocol was comparable among the three groups. The disease activity at year 2 followed a similar pattern when comparing the MTX monotherapy and TwHF monotherapy groups (Table 1). The TwHF was not inferior to MTX in treating active RA. However, the combination therapy was not obviously more effective than MTX monotherapy at year 2. This may suggest that the combination therapy induces disease remission faster in the early stage of treatment, while the long-term efficacy was similar to that in the monotherapy groups. The limited sample size available is a concern at this stage and may have caused bias in the efficacy evaluation.

Aside from the clinical efficacy measures, we also obtained and scored paired radiological images of the hands and wrists from these patients to further objectively validate the efficacy of treatment. Consistent with previous studies [25], radiological progression was associated with disease activity measured by change in the DAS28 (Table 4). This confirmed the functional relevance of the radiological evaluation [26]. Radiographic progression was comparable among the three groups, though the annual radiographic progression trended toward being smaller in the combination group.

**Table 4:** Changes in JE, JSN and mTSS by changes in DAS28 tertiles over 2 years

	ΔDAS28			p
	<2.02, *n* = 36	2.02–3.51, n = 36	>3.51, n = 37	
ΔJE	2.72 (5.08)	0.85 (2.00)	0.76 (1.40)	0.020
	1.02 (0.09, 2.53)	0.00 (0.00, 0.89)	0.00 (0.00, 1.26)	
ΔJSN	2.66 (4.03)	0.87 (1.77)	1.23 (2.52)	0.029
	0.65 (0.00, 4.27)	0.00 (0.00, 1.16)	0.00 (0.00, 0.92)	
ΔmTSS	5.36 (8.20)	1.72 (2.86)	2.04 (3.29)	0.009
	2.07 (0.72, 8.13)	0.71 (0.00, 2.18)	0.52 (0.00, 2.67)	

*JE* joint erosion, *JSN* joint space narrowing, *mTSS* modified total Sharp score, *DAS28* 28-joint count Disease Activity Scoreg

Data are presented as mean (standard deviation, SD) and median (interquartile range, IQR)

The *p* values were calculated using analysis of covariance with treatment as a covariate

Similar to the previous report, the safety profile showed that the frequency of adverse events was not significantly different among the three groups (Table 5). At year 2, the majority of patients who withdrew from the study did not do so in relation to adverse events. The antifertility effect of TwHF was well-known to the participants in our study, and the women recruited were mainly postmenopausal or were not planning to become pregnant. We monitored irregular menstruation in these women, and the incidence was similar among the three groups.

**Table 5:** Adverse events in patients

	MTX (n = 69)	TwFH (n = 69)	TwFH + MTX (n = 69)
All	45 (65)	33 (48)	35 (51)
Nausea	18 (26)	9 (13)	17 (25)
Diarrhoea	3 (4)	2 (3)	1 (1)
Abdominal discomfort	13 (19)	9 (13)	11 (16)
Liver dysfunction	13 (19)	5 (7)	8 (12)
Serious Infection	5 (7)	0(0)	2 (3)
Baldness	3 (4)	2 (3)	5 (7)
Ulcer	8 (12)	1 (1)	6 (9)
Irregular menstruation	5 (7)	7 (10)	5 (7)
Anemia	7 (10)	1 (1)	6 (9)
Leucocytopenia	4 (6)	2 (3)	5 (7)
Palpitations	3 (4)	0 (0)	1 (1)
Headache	3 (4)	1 (1)	2 (3)
Fatigue	5 (7)	2 (3)	1 (1)
Weight loss	3 (4)	0 (0)	1 (1)

*MTX* methotrexate, *TwHF Tripterygium wilfordii* Hook F

Data are presented as number (percentage)

We did not attribute the two cancer deaths to the use of TwHF in this follow-up study. A number of clinical studies on TwHF have not found any association between cancer and TwHF, but rather with RA per se [12, 13, 27]. Notably, the TwHF + MTX combination group in this follow-up study also received the same dosage of TwHF, yet experienced no malignancies. Moreover, numerous pharmacological studies have suggested triptolide has an anti-tumor effect in various tumor models in vitro and in vivo [28, 29].

This study, as a long-term extension of a 24-week RCT, has several limitations. First, this follow-up study, together with the TRIFRA study, was designed as an open-label study. To increase the objectiveness of the results, blinded evaluators were employed. However, a randomized double-blinded trial is needed to provide more robust data and confirm our results. Second, a significant proportion of patients were lost to follow up or changed to other regimens for different reasons. This could bias the analysis, and, therefore, the power of the conclusion was weakened (Fig. 1). Furthermore, there might be significant bias in the analysis of adverse events. In this case, we performed both ITT and PP analysis of all the clinical measures to determine whether similar patterns were observed. Importantly, it was noted that the proportions of patients who withdrew or were lost to follow up were similar among the three groups. It was presumed that patients returned to “real-world” clinical practice and their treatments were monitored and optimized by their physicians’ judgement. Thus, we compared the rate of adherence to the original protocol and performed detailed cause analysis in the follow up, and showed that these were comparable among the three groups. Third, only the radiographic images of the hands and wrists were available for this analysis, and the feet were not included. Studies have shown that the joints of the feet are usually affected earlier than the joints of the hands, and therefore including the feet could help improve the sensitivity of joint damage assessment in early RA [30, 31]. In order to demonstrate the therapeutic efficacy, the patients recruited in the TRIFRA study were diagnosed with definite active RA and the mean disease duration was longer than 60 months. Moreover, considerable radiographic damage was noted in the hands and wrists, providing a reasonable baseline background to determine slowing of radiographic damage, even though the feet were not evaluated. The scores for the hands and wrists were representative of the disease activity at this stage, which was also indicated by its association with change in the DAS28. Ideally, the radiographic data should be obtained uniformly at baseline and the end of year 2. In this real-world follow-up study, we only successfully performed radiographic examination at the end of the 2 years in a proportion of subjects. To further maximize the power of our results, the analysis included all the patients who had at least two radiographic examinations with time intervals greater than 1 year. Notably, we calculated the estimated yearly radiographic progression based on these images. This might introduce additional bias to our results because this model presumed that radiographic progression was linear. Finally, the dose of MTX was limited to 12.5 mg per week and the dose of folic acid was 10 mg per week, which is very common in Asia. This point was discussed in our previous report on the TRIFRA study. As a follow up of the TRIFRA study, the same doses of MTX and folic acid were maintained. Although there has not yet been a direct comparison of the response rates to MTX in patients with RA from different ethnicities, we did notice that the clinical response in our TRIFRA trial was comparable to the data from patients with RA in Europe and North America receiving a higher dose of MTX [32]. We recognized that the response rate in RA may be further improved with more intensive combination treatment [33], although the frequency of adverse events may also increase as well. Most reviews and recommendations suggest a prescription of at least 5 mg folic acid (FA) per week [34]. So far, insufficient evidence has been collected on the optimal dose of FA. Studies have proven that low-dose FA (≤ 7 mg/week) was able to reduce MTX side effects significantly [35]. Doses larger than 5 mg did not lead to further amelioration of the MTX side effects and neither did they affect the efficacy of MTX therapy [36, 37]. Because of these considerations, we are less concerned that the therapeutic effects of MTX were compromised by our use of FA in our study.

This study confirmed the original finding that TwHF was not inferior to MTX in treating active RA in the long term, and that a further study with larger sample sizes should be performed to characterize the role of TwHF in RA therapy in greater detail.

## Conclusions

Although this is a follow-up study with several limitations, including the relatively high rates of withdrawal and treatment strategy modifications, data from both the ITT and PP analyses indicate that TwHF monotherapy was not inferior to MTX monotherapy in controlling disease activity and retarding radiographic progression in patients with active RA. This is consistent with the previously published, randomized, controlled, parallel arm, active-comparator TRIFRA study.

## Abbreviations

ACR: American College of Rheumatology
cDAI: Clinical Disease Activity Index
CRP: C-reactive protein
DAS28: 28-joint count Disease Activity Score
DMARD: Disease-modifying antirheumatic drug
ESR: Erythrocyte sedimentation rate
FA: Folic acid
HAQ: Health Assessment Questionnaire
ITT: Intent-to-treat
JE: Joint erosions
JSN: Joint space narrowing
LDA: Low disease activity
LOCF: Last observation carried forward
mTSS: Modified total sharp score
MTX: Methotrexate
PP: Per-protocol
PUMCH: Peking Union Medical College Hospital
RA: Rheumatoid arthritis
RCT: Randomized controlled trial
SDD: Smallest detectable difference
SF-36: 36-item Short-Form Health Survey questionnaire
SJC: Swollen joint count
TJC: Tender joint count
TwHF: Tripterygium wilfordii Hook F
VAS: Visual analog scale
VFE: Valid-for-efficacy
